# Robust Pt/Au Composite Nanostructures for Abiotic Glucose Sensing

**DOI:** 10.3390/bios15090588

**Published:** 2025-09-08

**Authors:** Asghar Niyazi, Ashley Linden, Mirella Di Lorenzo

**Affiliations:** 1 Department of Chemical Engineering, University of Bath, Bath BA2 7AY, UK; an993@bath.ac.uk (A.N.); alinden@guilford.edu (A.L.); 2 Centre for Bioengineering and Biomedical Technologies (CBio), University of Bath, Bath BA2 7AY, UK; 3 Institute for the Augmented Human, University of Bath, Bath BA2 7AY, UK; 4 Institute of Sustainability and Climate Change, University of Bath, Bath BA2 7AY, UK

**Keywords:** highly porous gold, polyaniline, platinum nanoparticles, glucose, electrochemical biosensor

## Abstract

Effective glucose monitoring is paramount for patients with diabetes to effectively manage their condition and prevent health complications. Electrochemical sensors for glucose monitoring have key advantages over other systems, including cost-effectiveness, miniaturisation and portability, enabling the design of compact and wearable devices. Typically, enzymes are used in these sensors, with the limitations of poor stability and high cost. In alternative, this study reports the development of a gold and platinum composite nanostructured electrode and its testing as an abiotic (enzyme-free) electrocatalyst for glucose oxidation. The electrode consists of a film of highly porous gold electrodeposited onto gold-plated electrodes on a printed circuit board (PCB), which is coated with polyaniline decorated with platinum nanoparticles. The resulting nanocomposite structure shows a sensitivity towards glucose as high as 95.12 ± 2.54 µA mM^−1^ cm^−2^, nearly twice that of the highly porous gold electrodes, and excellent stability in synthetic interstitial fluid over extended testing, thus demonstrating robustness. Accordingly, this study lays the groundwork for the next generation of durable, selective, and affordable abiotic glucose biosensors.

## 1. Introduction

Diabetes mellitus, a chronic disease caused by insufficient insulin production or ineffective insulin utilisation, has become a severe global public health issue. Effective management of this condition requires regular monitoring of blood glucose levels, which, in healthy individuals, should be maintained between 3.9 and 7 mM before meals, and below 7.8 mM post-meals [1,2]. Failure to properly manage diabetes can result in complications such as neuropathy, retinopathy, cardiovascular diseases, kidney failure, and even mental health issues [1,3]. With over 463 million people affected worldwide in 2019, and projections estimating this figure will rise to 700 million by 2045, the need for precise, accessible, and reliable glucose monitoring systems is more urgent than ever [4].

Over the past two decades, the introduction of continuous glucose monitoring systems has dramatically changed diabetes care, providing patients with the ability to track their glucose levels in real-time. Devices that measure glucose concentrations from alternative biofluids such as sweat, saliva, and interstitial fluid offer the significant advantage over traditional blood-based methods of being non-invasive, and integration with mobile apps enables seamless monitoring, empowering patients to manage their condition more effectively [5,6].

Typically, the enzyme glucose oxidase is used as the primary catalyst in glucose biosensors, leading to remarkable selectivity and sensitivity as high as 97.34 μA mM^−1^ cm^−2^ [7]. However, enzymatic sensors face limitations such as susceptibility to environmental factors like pH, temperature, and humidity, as well as challenges with long-term stability and cost-effectiveness [8]. Non-enzymatic, abiotic glucose biosensors, by contrast, have gained significant attention for their simpler preparation processes, superior long-term durability, and ability to overcome the inherent limitations of enzymatic sensors. These devices rely on the direct abiotic electrochemical oxidation of glucose on electrode surfaces, making them more suitable for repeated use and diverse operating environments [8,9].

Recent advancements in non-enzymatic glucose sensors have leveraged noble metals like gold and platinum, both exhibiting exceptional electrocatalytic activity and sensitivity towards glucose oxidation at the nanoscale [10,11]. Several nanostructures of these metals have been investigated for glucose electro-oxidation, including nanoparticles, nanorods, porous films, and nanowires, each offering a high specific surface area and active sites for catalysis [1]. In particular, porous gold structures have been extensively studied for glucose electro-oxidation, including as the anode catalyst in glucose fuel cells (GFCs), achieving a current density of approximately 32 µA cm^−2^ in phosphate buffer containing 6 mM glucose [12,13]. Pt nanoparticles are often combined with other metals, such as gold, to improve performance. For example, Mahobiya et al. used Au-Pt nanostructures for glucose detection, achieving a very low limit of detection, 0.1 nM, and high sensitivity (0.31 μA nM^−1^) [14]. To prevent Pt particles from leaching and/or aggregating during operation, several strategies have been proposed to enhance stability, including the use of cellulose nanocrystals as a framework for Pt nanoparticles dispersity [15]. More generally, conductive polymers have been explored as a way to stabilise metal nanostructures without compromising the electron transfer processes. Amongst the several options, polyaniline (PANI) shows promising features, such as excellent electrical conductivity and ease of synthesis, which make this polymer an ideal candidate for the mechanical stability of nanostructured metal catalysts while maintaining electrical performance [16,17,18].

In this study, we explore the use of Pt/Au composite nanostructures on printed circuit boards (PCBs) for glucose detection. The nanostructures consist of a film of highly porous gold (hPG) electrodeposited on gold-plated electrodes on a PCB, which is coated with a layer of PANI decorated with platinum nanoparticles. A novel one-step approach is used, which, compared to a traditional sequential method that would imply separate steps to synthesise PANI and subsequently deposit Pt nanoparticles [19,20,21], offers the advantages of a faster synthesis process and lower costs as one solution is used. Through our approach, the Pt nanoparticles are embedded within the PANI structure, which helps preventing the leaching of the nanoparticles and enhances the overall stability of the resulting composite electrode. The synthesised electrodes are tested for the abiotic detection of glucose in both phosphate buffer and synthetic interstitial fluid, and performance is compared with that of bare hPG electrodes and hPG electrodes coated with PANI but without Pt nanoparticles. Stability is assessed via repetitive testing of the electrodes over time. The facile and fast approach used for the electrodes manufacturing, which integrates in one step the electropolymerisation of PANI together with the electrodeposition of Pt nanoparticles, enables cost-effective mass production, while the use of PCBs facilitates electronic integration for GFC design. Accordingly, this study provides a robust and cost-effective alternative for abiotic glucose monitoring in interstitial fluid.

## 2. Materials and Methods

### 2.1. Reagents and Apparatus

All chemicals used were of analytical grade and employed as received without further modification unless specified otherwise. Aqueous solutions were prepared using ultrapure water (18.2 MΩ cm^−1^) obtained from a Milli-Q system (Millipore, UK).

Potassium chloride (KCl), sodium phosphate dibasic (Na_2_HPO_4_), sodium phosphate monobasic (NaH_2_PO_4_), and hydrogen tetrachloroauric acid (HAuCl_4_) were sourced from Alfa Aesar. α-D(+)-glucose was purchased from Fisher Scientific (Leicestershire, UK). Sulfuric acid (H_2_SO_4_), ammonium chloride (NH_4_Cl), aniline, chloroplatinic acid solution (8 wt. % in H_2_O) (H_2_PtCl_6_), perchloric acid (HClO_4_), calcium chloride (CaCl_2_), HEPES sodium salt, potassium ferricyanide, sodium chloride (NaCl), magnesium sulphate (MgSO_4_), lactic acid, ascorbic acid, uric acid, acetaminophen, sucrose, and l-cysteine, were procured from Sigma Aldrich (Poole, UK).

### 2.2. Experimental Conditions

All experiments were conducted in air-saturated 0.1 M phosphate buffer (PB) at pH 7.4, which was prepared by dissolving Na_2_HPO_4_ and NaH_2_PO_4_ in ultrapure water. A 2 M stock solution of D(+)-glucose in PB was prepared and left at room temperature overnight to allow mutarotation from the α- to β-monomer prior to use [12].

Synthetic Interstitial Fluid (SIF) was prepared as per [22]. This solution consisted of 2.5 mM CaCl_2_, 10 mM HEPES sodium salt, 3.5 mM KCl, 0.7 mM MgSO_4_, 123 mM NaCl, 1.5 mM NaH_2_PO_4_, and 7.4 mM sucrose in PB. The pH was adjusted to 7.4 by adding an appropriate amount of 1 M HCl. SIF was stored in the dark at room temperature when not in use.

### 2.3. Fabrication of the hPG/Au, PANI/hPG/Au, and PANI-Pt/hPG/Au Electrodes

All electrochemical tests were performed by using either Autolab PG302 N or PalmSens4 potentiostats, with data logging and analysis managed through NOVA and PSTrace software, respectively. The electrodes were connected to the potentiostat via a Peripheral Component Interconnect (PCI) sourced from RS Components (UK). Cleaning and activation of the Au electrodes on PCB were carried out prior to testing by sweeping the potential between 0 V and +1.6 V (vs. Ag/AgCl, 3M KCl) over 12 cycles in 0.05 M H_2_SO_4_ at a scan rate of 100 mV s^−1^. A three-electrode system was used, comprising an Ag/AgCl (3M KCl) reference electrode and a platinum wire counter electrode (0.5 mm diameter, from Alfa Aesar, UK).

**Figure 1:**
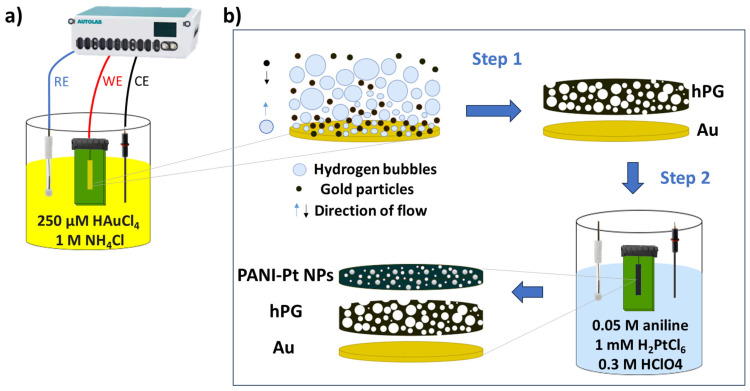
Fabrication of electrodes on gold-plated electrodes on a printed circuit board. (**a**) Electrochemical set-up for hPG deposition. (**b**) Steps for the electrode functionalisation. A film of highly porous gold (hPG) is deposited via the dynamic hydrogen bubble template. The concurrent electro-polymerisation of polyaniline (PANI) and electrodeposition of Pt nanoparticles onto the hPG surface follows.

Figure 1 shows the steps followed for the synthesis of the nanostructured composite electrodes used in this study. A hPG film was electrodeposited onto the gold-plated (Au) electrodes on PCB via the dynamic hydrogen bubble template previously described [23]. Briefly, the Au electrodes were immersed in a solution comprising 250 µM HAuCl_4_ and 1 M NH_4_Cl, and a current of −320 mA was applied for 180 s with the aforementioned three-electrode configuration. Subsequently, the electrodes were thoroughly washed with Milli-Q water and activated via cyclic voltammetry (CV) in 0.05 M H_2_SO_4_, with the potential swept between 0 V and 1.6 V for five cycles versus Ag/AgCl (3M KCl) at a scan rate of 50 mV s^−1^.

Electropolymerisation of PANI onto the hPG/Au electrodes was carried out via chronopotentiometry (CP), at a constant current of 20 µA for 20 min in an electrolyte consisting of 0.05 M aniline and 0.3 M HClO_4_, leading to the PANI/hPG/Au electrodes. To embed Pt nanoparticles within the polymeric structure, the formation and deposition of Pt nanoparticles were concurrent with PANI electrodeposition by adding 1 mM H_2_PtCl_6_ in the electrolyte, as previously described [23].

### 2.4. Material Characterisation

The electroactive surface area (*ESA*) of hPG/Au was evaluated by cyclic voltammetry (CV) in 0.05 M H_2_SO_4_, over a potential range of 0–1.6 V versus Ag/AgCl (3 M KCl), with a scan rate of 50 mV s^−1^ for five cycles. *ESA* was calculated by considering the charge of the electrode, which was obtained by integrating the peak area of the gold oxide reduction and dividing it by the scan rate, according to Equation (1):

(1) $$ESA\;=\;\frac{Q}{386}$$

Where *Q* is the charge of the electrode (μC), and 386 μC cm^−2^ is the estimated charge for a 1 cm^2^ polycrystalline Au electrode [12]. This method could not be applied to the composite electrodes (PANI/hPG/Au and PANI-Pt/hPG/Au), as the PANI and Pt nanoparticles introduced additional faradaic currents that interfered with accurate peak integration. The surface morphology of all electrodes was examined by Field Emission Scanning Electron Microscopy (FESEM) using a JEOL JSM-6480LV instrument (JEOL Ltd., Tokyo, Japan). Energy-dispersive X-ray spectroscopy (EDX) and elemental mapping were performed with a Scanning Electron Microscope (SEM, JEOL JSM-6480LV; JEOL Ltd., Tokyo, Japan), equipped with an energy-dispersive X-ray spectroscopy detector. For additional structural characterisation, the electrodes were studied using Transmission Electron Microscopy (TEM) on a JEOL JEM-2100Plus instrument (JEOL Ltd., Tokyo, Japan). FESEM, SEM, and TEM images were analysed with the ImageJ software, version 1.53t.

### 2.5. Assessing the Performance of the Nanocomposite Electrodes

Electrochemical impedance spectroscopy (EIS) and CV were employed to assess the charge transfer resistance and electrochemical characteristics of the electrodes generated. The experiments were carried out in 0.1 M PB containing 6 mM glucose, and the electrodes were tested in the three-electrode set-up described above. EIS was measured across a frequency range of 0.1 Hz to 100,000 Hz, using a 10 mV AC perturbation, while CV was recorded over a potential range of 0 to 1 V versus Ag/AgCl (3M KCl) at a scan rate of 5 mV s^−1^.

The electrochemical response to glucose of the electrodes was assessed by chronoamperometry (CA), conducted in 0.1 M PB (pH 7.4) under different concentrations of glucose, ranging from 0 to 100 mM, in the three-electrode set-up described above, for a total duration of 300 s. CA measurements were carried out at the potential corresponding to the glucose oxidation peak observed in preliminary CV analyses. In particular, this potential resulted to be: +0.24 V vs. Ag/AgCl (3M KCl) for hPG/Au; and +0.29 V vs. Ag/AgCl (3M KCl) for PANI-Pt/hPG/Au and PANI/hPG/Au.

In the case of PANI-Pt/hPG/Au, tests were also performed in SIF containing glucose at a concentration within the range of 0–25 mM.

The sensitivity, *s* (µA mM^−1^ cm^−2^), towards glucose was calculated from the slope of the initial linear range of the calibration curve obtained by CA tests in PB as:

(2) $$s\;=\;\frac{∆i}{∆c}$$

Where *i* is the current density (µA cm^−2^) and *c* is glucose concentration (mM).

The sensitivity towards glucose of PANI-Pt/hPG/Au was also evaluated in SIF.

The low limit of detection (*LOD*) was assessed by measuring the standard deviation (*σ*) of the current in PB solution without glucose, as follows:

(3) $$LOD\;=\;\frac{3\sigma }{s}$$

The shelf-life stability of PANI-Pt/hPG/Au was assessed in both PB and SIF containing 6 mM glucose by performing repetitive CA measurements on the same electrode over a period of 90 days. In between measurements, the electrodes were stored at room temperature and left open-air without specific precautions.

## 3. Result and Discussion

### 3.1. Physical Characterisation

**Figure 2:**
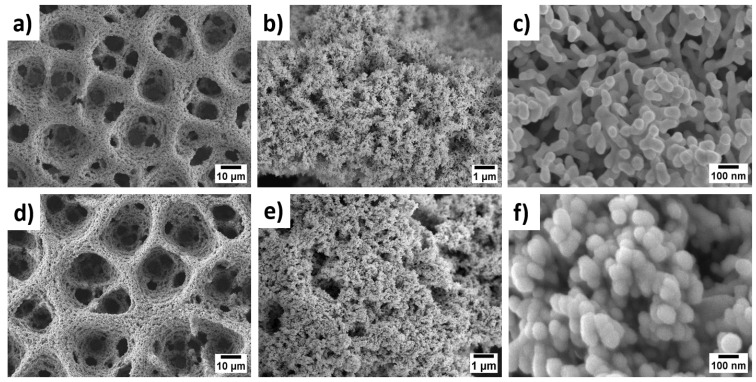
FESEM images at increasing magnifications: ×1000, ×10,000, and ×100,000, from right to left, respectively, of: (**a**–**c**) hPG/Au electrode and (**d**–**f**) PANI-Pt/hPG/Au. All images were captured at an acceleration voltage of 5.0 kV.

The surface architecture of PANI-Pt/hPG/Au was examined by FESEM and TEM and compared with hPG/Au and PANI/hPG/Au. As shown in Figure 2, hPG/Au displays a three-dimensional porous framework with uniformly distributed macropores (~10 µm), interconnected through a nanostructured gold network with a coral-like appearance. This hierarchical porosity, created by hydrogen bubble templating during electrodeposition [13], greatly increases the electroactive surface area (ESA), reaching 24.8 cm^2^ compared to only 0.072 cm^2^ for flat gold electrodes (Figure S1). Such a large ESA is beneficial for glucose oxidation because it provides abundant active sites and facilitates mass transport of analyte molecules. At higher magnification, the hPG surface appears densely packed with dumbbell-shaped nanostructures (Figure 2b,c) [24].

**Figure 3:**
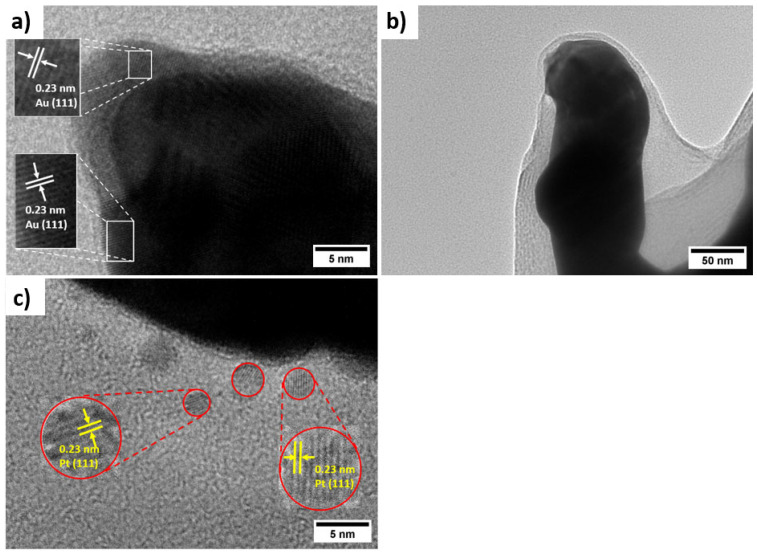
Characterisation of the morphology of the nanocomposite electrodes: High-magnification TEM micrographs of (**a**) hPG/Au, (**b**) PANI/hPG/Au, and (**c**) PANI-Pt/hPG/Au.

As shown in Figure 2d–f, the electropolymerisation of PANI with simultaneous incorporation of Pt nanoparticles preserves the porous framework of hPG/Au. However, the pore walls become thicker, and the surface develops a granular, cauliflower-like nanostructure, composed of compact nanoparticles with an average size of 74 ± 11 nm. This nanostructure is expected to enhance catalytic activity by exposing Pt/PANI sites to glucose molecules while maintaining efficient pathways for electron transfer. The FESEM of PANI/hPG/Au (Figure S2) shows a similar nanostructure, thus confirming that the loading of Pt nanoparticles alters only the surface roughness but preserves the underlying porosity. TEM further confirmed the hierarchical structure of these electrodes (Figure 3). The hPG film exhibited crystalline features with an interplanar spacing of 0.23 nm, consistent with the (111) planes of an FCC lattice [25]. When coated with PANI or PANI-Pt, hPG was accordingly covered either with a protective polymeric film only (Figure 3b) or with a uniformly distributed layer of Pt nanoparticles, with a size ranging from 3.5 to 14.6 nm, embedded within the protective polymer matrix (Figure 3c).

The functionalization of hPG/Au with a coating of either PANI only or leads to a protective layer (Figure 3b) and in the case of PANI decorated with Pt nanoparticles, a uniform dispersion of Pt nanoparticles is observed, with sizes ranging from 3.5 nm to 14.6 nm, depending on whether they are embedded within or on the surface of the PANI structure (Figure 3c).

**Figure 4:**
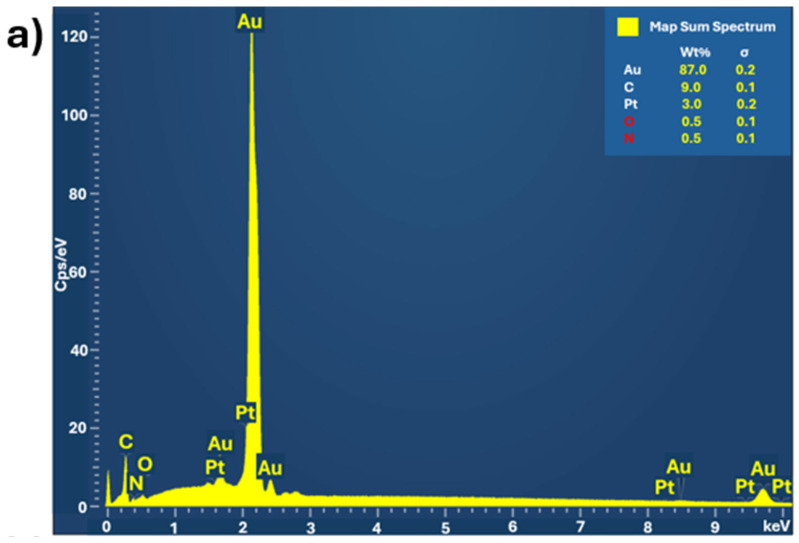

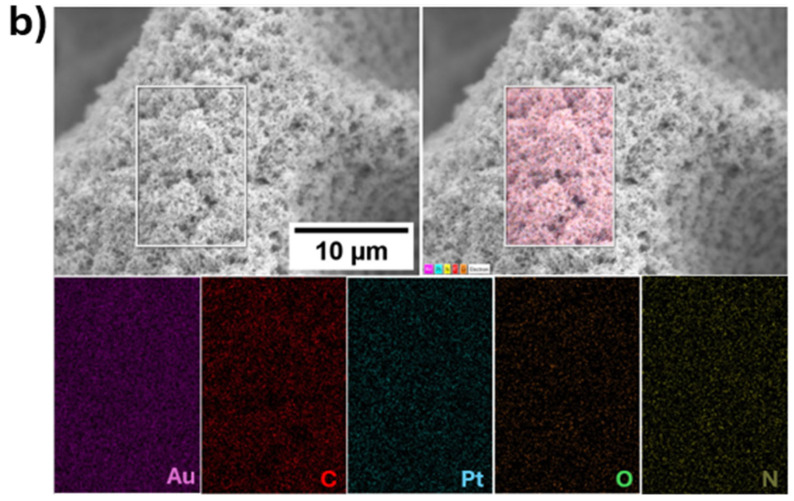
(**a**) EDX spectrum of PANI-Pt/hPG/Au showing the elemental composition, and (**b**) Elemental mapping of the same region of PANI-Pt/hPG/Au, confirming the uniform distribution of elements across the composite surface.

All electrodes exhibit a high surface-to-volume ratio. The structure, crystallinity, and elemental composition of hPG/Au and PANI-Pt/hPG/Au were characterised using XRD, SAED, Raman spectroscopy, and XPS to verify the formation of the desired nanocomposite structures. XRD and SAED confirmed the face-centred cubic (FCC) lattice of Au in hPG and the crystalline nature of Pt nanoparticles within the polymer matrix, with characteristic reflections for Pt (111), (002), (022), and (113) planes [26]. Raman spectroscopy revealed the formation of conductive PANI in its emeraldine salt form and indicated modifications in the quinoid/benzenoid ratio upon Pt incorporation, consistent with interactions between Pt nanoparticles and PANI. XPS analysis showed the presence of metallic Au^0^ and Pt^0^ as dominant species, with minor Pt^2+^ contributions, confirming successful incorporation of Pt nanoparticles and the chemical stability of the composite. A comprehensive surface characterisation, reported in a previous study [23], confirms the successful formation, reproducibility, and structural integrity of the PANI-Pt/hPG/Au nanocomposite. EDX and elemental mapping were also performed on a small area of the surface of PANI-Pt/hPG, as shown in Figure 4. The EDX analysis (Figure 4a) revealed that the electrode predominantly consisted of gold (87%), as expected, considering the hPG film. Carbon was also present at 9%, which can be attributed to the PANI component of the composite. Pt, introduced as nanoparticles, accounted for approximately 3% of the elemental composition. Small amounts of oxygen and nitrogen, each contributing around 0.5%, were also detected, likely originating from the PANI polymeric matrix and possible surface oxidation [27,28]. Elemental mapping further confirmed a uniform and distinctive spatial distribution of the elements across the scanned area, indicating successful deposition and good integration of the PANI-Pt layer onto the hPG framework.

### 3.2. Electrochemical Characterisation of the Electrodes

**Figure 5:**
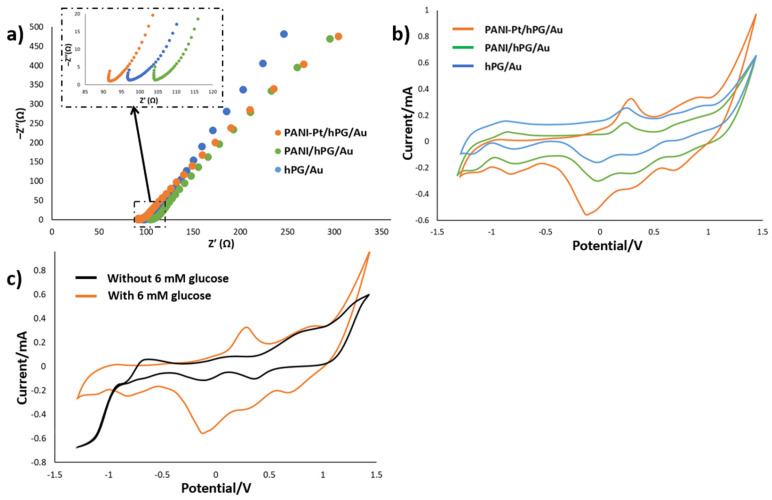
Electrochemical characterisation of hPG/Au, PANI/hPG/Au, and PANI-Pt/hPG/Au in PB containing 6 mM glucose: (**a**) Nyquist plots from EIS showing charge transfer resistance, with the inset depicting a zoom-in of the initial regions of the same electrodes; (**b**) Cyclic voltammograms at a scan rate of 5 mV s^−1^; and (**c**) Cyclic voltammograms of PANI-Pt/hPG/Au in PB with and without 6 mM glucose. Measurements were performed in a three-electrode set-up with an Ag/AgCl (3M KCl) reference electrode and a platinum wire counter electrode.

The intrinsic electrochemical behaviour of PANI-Pt/hPG/Au was studied by CV and EIS, and the results were compared with hPG/Au and PANI/hPG/Au. The EIS spectra (Figure 5a) show that hPG/Au has a relatively low initial impedance, indicating good electrical conductivity. As expected, the polymer coating, leading to PANI/hPG/Au, increases the impedance due to reduced conductivity from the polymer layer. Nonetheless, decorating PANI with Pt nanoparticles, PANI-Pt/hPG/Au electrode, greatly lowers the impedance, giving the lowest value among the three electrodes. This result is attributed to the enhanced conductivity and catalytic activity of Pt nanoparticles. Despite these differences, both PANI/hPG/Au and PANI-Pt/hPG/Au show similar slopes at low frequency, indicating that the diffusion-controlled processes within their porous structures are comparable.

CV measurements (Figure 5b) reveal an oxidation peak between 0.2 and 0.3 V versus Ag/AgCl (3 M KCl) for all three electrodes, which corresponds to glucose oxidation. In particular, this oxidation peak occurs at the potential of 0.29 V for PANI-Pt/hPG/Au, 0.25 V for hPG/Au, and 0.26 V for PANI/hPG/Au, with corresponding currents, respectively, of 0.32, 0.26, and 0.14 mA. The slightly higher peak potential, and significantly greater current in the case of PANI-Pt/hPG/Au, suggest that embedding Pt nanoparticles within the PANI layer provides a synergic catalytic effect together with hPG, which enhances glucose oxidation and facilitates charge transfer at the electrode interface [23]. hPG/Au also shows great catalytic activity, due to its high surface area and porosity that promote high reaction kinetics [29]. By contrast, PANI/hPG/Au exhibits the lowest output current, likely because of the lower conductivity of PANI compared to noble metals, which limits electron transfer [30].

Despite these differences in current, the similarity in peak potentials confirms that glucose oxidation occurs on all surfaces. Variations in peak shape and height reflect differences in catalytic efficiency and charge transfer kinetics, as shown by Neha et al. [31], who demonstrated that while Pt and Au nanoparticles both catalyse glucose oxidation, their reaction pathways and intermediate species differ, affecting the current response and peak morphology. Furthermore, CVs of PANI-Pt/hPG/Au recorded with and without 6 mM glucose (Figure 5c) show no distinct oxidation peak between 0.2 and 0.3 V in PB, confirming that the observed peaks are due to glucose oxidation. Collectively, these results emphasise the role of electrode composition and surface modification in enhancing charge transport and catalytic performance for glucose electro-oxidation [32,33].

**Figure 6:**
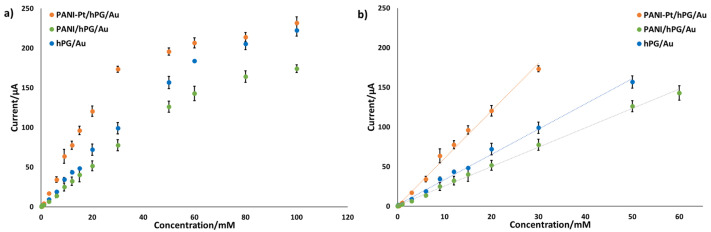
Amperometric response of hPG/Au, PANI/hPG/Au, and PANI-Pt/hPG/Au to increasing concentrations of glucose in PB. (**a**) response to glucose varying from 0 to 100 mM; (**b**) linear range response. Error bars represent the SD from a minimum of three independent measurements (n ≥ 3) using different electrodes.

Figure S3a–c show the chronoamperometric response of hPG/Au, PANI/hPG/Au, and PANI-Pt/hPG/Au to varying concentrations of glucose in PB. As shown in Figure 6, each electrode exhibited two distinct linear regions in its calibration curves, which correspond to low- and high-concentration ranges of glucose. The first linear region, at low glucose concentrations, reflects a regime where the reaction rate is directly proportional to glucose availability. At higher concentrations, active sites on the electrode surface become saturated, which slows the rate of increase in current and changes the sensitivity of the electrode, as previously observed [34,35]. The first linear zone includes clinically relevant values of glucose [2], so it was the region considered for assessing the sensitivity of the electrodes.

PANI-Pt/hPG/Au exhibited the largest sensitivity, with a value of 95.12 ± 2.54 µA mM^−1^ cm^−2^ up to a glucose concentration of 30 mM, and the lowest detection limit of 0.047 mM. hPG/Au showed a sensitivity towards glucose of 50.63 ± 1.05 µA mM^−1^ cm^−2^ with an LOD of 0.060 mM over the range 0.1–50 mM. PANI/hPG/Au exhibited the lowest sensitivity of 38.93 ± 0.58 µA mM^−1^ cm^−2^ up to 60 mM glucose, with the highest LOD of 0.202 mM.

**Figure 7:**
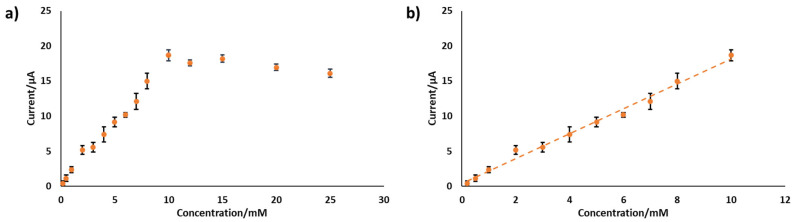
Amperometric response of PANI-Pt/hPG/Au to increasing concentrations of glucose in SIF. Data are the average from three replicate measurements obtained with three different electrodes. (**a**) response to glucose varying from 0 to 25 mM; (**b**) linear range response. Error bars represent the SD from a minimum of three independent measurements using different electrodes.

The best-performing electrode, PANI-Pt/hPG/Au, was tested in SIF, and the results are shown in Figure 7. In this solution, the electrode showed a sensitivity of 28.23 ± 2.39 µA mM^−1^ cm^−2^ up to 10 mM glucose, a sensitivity more than three times lower than in PB. The amperometric response of this electrode to glucose concentrations above 10 mM remained unchanged, with a slight decrease. This behaviour can be attributed to the more complex ionic composition of SIF compared with PB, where multiple ions compete with glucose in the interaction with the electrode surface, thus affecting electron transfer and overall reducing the current output. By-products from reactions may cause fouling of the electrode surface, further limiting mass transport and affecting the sensor’s responsiveness [22,36]. In particular, Cl^−^ can adsorb within the pores of nanostructured Pt and Au, partially poisoning the catalytic sites and slowing glucose oxidation. For instance, our team has previously shown that in the presence of 5 mM Cl^−^, hPG electrodes showed a reduction in current output of approximately 20.2% [30]. Non-specific adsorption of ions at the electrode interface can significantly hinder electron transfer, particularly in systems based on PANI and metal catalysts. This happens because adsorbed ions may compete with glucose at the active sites or disturb the electrical double layer, thereby altering the interfacial environment required for effective charge transfer [37,38]. Cations such as Ca^2+^, which are present in SIF, are especially prone to these effects. Their interaction with the PANI–metal boundary can destabilise the electrode potential [39], leading to reduced sensitivity and lower reliability of electrochemical measurements. Similar phenomena have also been observed in carbon- and metal-doped PANI composites, where the surrounding ionic environment strongly affects redox behaviour and charge transfer efficiency [40].

The performance of PANI-Pt/hPG/Au was compared with abiotic glucose sensors previously reported. As summarised in Table 1, PANI-Pt/hPG/Au shows a wider detection range and excellent sensitivity in both SIF and PB, confirming its potential for practical application.

### 3.3. Assessing the Stability of the Electrodes in PB and SIF

**Figure 8:**
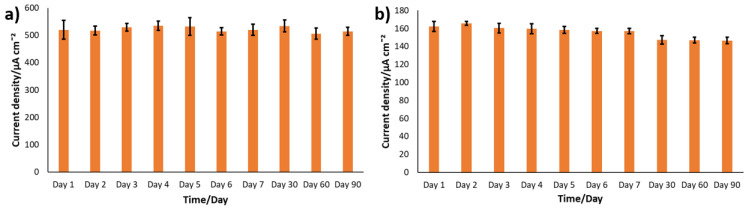
Assessment of the stability of PANI-Pt/hPG/Au under repetitive CA testing in either (**a**) 0.1 M PB or (**b**) SIF, both containing 6 mM glucose. Current density was calculated by referring to the anode’s geometric area (0.0628 cm^2^). The repetitive tests were carried out using the same electrochemical cell, and each data point represents the mean of three replicates. After each test, the electrodes were rinsed thoroughly with deionised water, air-dried, and kept at room temperature. Error bars show the standard error from a minimum of three independent measurements (n ≥ 3) using different electrodes.

Besides showing good sensitivity, a sensor should also demonstrate strong long-term stability. A particular challenge in enzyme-based sensors is the shelf-life. Accordingly, the shelf-life of the nanostructured electrodes developed in this study was assessed by CA at a constant glucose concentration of 6 mM, over a period of three months. Data were collected daily for the first seven days, and subsequently after one, two, and three months. After each test, the electrodes were rinsed thoroughly and stored without any specific storage precaution. As reported in Table 2 and shown in Figure 8, both PANI-Pt/hPG/Au and PANI/hPG/Au exhibited excellent stability, maintaining a high level of electrochemical activity throughout the three months. PANI acts as a protective layer for hPG, which is otherwise fragile and tends to detach from the surface over time [30]. Not only does PANI shield hPG with its mechanical strength, but it also prevents Pt nanoparticles from agglomerating by keeping them well dispersed, resulting in excellent overall stability [46]. In fact, in the absence of PANI, hPG/Au demonstrated an over 85% decline in the current, from 24.29 µA (386.89 µA cm^−2^) to 3.59 µA (57.3 µA cm^−2^), as shown in Figure S4.

The shelf-life of PANI-Pt/hPG/Au was assessed in SIF as well. As shown in Figure 8, in SIF PANI-Pt/hPG/Au, it retained most of its activity, with current declining from 162.14 ± 5.57 µA cm^−2^ on day 1 to 146.43 ± 3.66 µA cm^−2^ on day 90, which corresponds to an overall decrease of approximately 9.6%.

Table 3 compares the stability observed in this work with other studies taken from recent literature. The stability of PANI-Pt/hPG/Au in SIF after three months of testing is the largest so far reported in a complex medium. While many studies focus on short-term tests in buffer solutions that do not contain electrochemically interfering compounds, this work stands out by proving a remarkable stability over time in more realistic conditions, which is greatly promising for practical uses. In one study [46], a NiFe-PANI catalyst retained a significant portion of its initial performance after one month of testing. In comparison, our catalyst demonstrated even better stability over a longer period (more than three months). This improved performance may be due to structural differences arising from the different deposition method we used, which, in contrast to the sequential deposition used in the study, creates a layer of PANI embedding Pt nanoparticles in a single step. The resulting structure provides conductive pathways throughout the nanoparticles and acts as a glue, preventing nanoparticle detachment and resulting in longer-lasting stability. Future work will focus on integrating PANI-Pt/hPG/Au into a fuel cell-based wearable device and on assessing long-term biocompatibility.

## 4. Conclusions

Abiotic catalysts for glucose electro-oxidation can help overcome major issues associated with the use of enzymes in sensors, namely, high cost and poor stability. Accordingly, these catalysts can help generate enhanced sensors for glucose monitoring systems, ultimately for the benefit of those patients who need to accurately control the level of glucose in their bodies. This work concerns the development of a composite nanostructured electrode on PCB, PANI-Pt/hPG/Au, which consists of a film of highly porous gold onto Au-plated electrodes, coated with a layer of PANI decorated with Pt nanoparticles, and demonstrates its use as a glucose sensor. Performance is assessed in terms of sensitivity, low limit of detection and shelf-life, and compared with the cases of the hPG/Au and PANI/hPG/Au electrodes.

As expected, amongst the three types of electrodes tested, PANI-Pt/hPG/Au exhibited superior performance, achieving high sensitivity and broad detection ranges in both PB and SIF, the latter simulating the complexity of a real biological fluid considered for wearable and minimally invasive applications. Specifically, PANI-Pt/hPG/Au demonstrated a sensitivity of approximately 28.23 ± 2.39 µA mM^−1^ cm^−2^ in SIF within the clinically relevant glucose concentration range of 2–7 mM. Importantly, PANI-Pt/hPG/Au exhibited excellent stability over time, with a retention of over 90% of the initial activity in SIF, thereby confirming its robustness under physiologically relevant conditions.

These findings highlight the strong potential of PANI-Pt/hPG/Au for reliable glucose monitoring in interstitial fluid. Overall, this study lays a solid foundation for the next generation of non-enzymatic glucose biosensors, offering a promising route towards a more durable, selective, and cost-effective solution.

## Figures and Tables

**Table 1:** The glucose sensitivity of abiotic nanostructured electrodes recently reported in the literature.

Working Electrode Catalyst	Linear Range	Solution	Sensitivity (μA mM^−1^ cm^−2^)	Ref.
hPG coated with PANI containing Pt nanoparticles	0.2–10 mM	SIF	28.23 ± 2.39	This work
hPG coated with PANI containing Pt nanoparticles	0.1–30 mM	0.1 M PB solution, pH 7.4	95.12 ± 2.54	This work
Gold Nanowires	0–1.15 mM	10 mM PB, pH 7.4	23.72	[41]
hPG	0.3–9 mM	0.1 M PB, pH 7.4	8.8	[12]
Highly porous platinum black on Au micro-needles electrode arrays	1–20 mM	10× PB, pH = 7.4	5.78 ± 0.17	[42]
Highly porous platinum black on Au micro-needles electrode arrays	1–20 mM	SIF	4.38 ± 0.21	[42]
Flexible AuNPs/PANI/carbon cloth integrated electrode	10.26 μM–10 mM	0.5 M KOH	150	[43]
Pt decorated PANI/polyvinyl alcohol hydrogel	1 μM–30 mM	0.1 M PB, pH 7.4	7.364	[44]
Nanofeather-like gold film	2.5–20 mM	0.1 M PBS solution, pH 7.4	9.6 ± 1.5	[45]

**Table 2:** Comparison of the shelf-life of several electrodes in PB after three months.

Electrode	Initial Current (µA cm^−2^)	Percentage Decrease (%)
hPG/Au	386.89 ± 29.14	85.2%
PANI/hPG/Au	247.68 ± 9.39	2.19%
PANI-Pt/hPG/Au	519.86 ± 35.03	3.94%

**Table 3:** Comparison of the stability of abiotic nanostructured electrodes recently reported in the literature.

Details on the Nanostructured Electrode	Stability Towards Glucose Oxidation	Testing Time and Conditions *	Ref.
PANI-Pt/hPG/Au	>96% retention of initial current	Repetitive CA tests in 6 mM glucose, 0.1 M PB, at 37 °C, over 3 months	This work
PANI-Pt/hPG/Au	>90% retention of initial current	Repetitive CA tests in 6 mM glucose in SIF, at 37 °C, over 3 months	This work
3D carbon nanocoils on hierarchical macroporous nickel foam/Cu@Ni core–shell nanoparticles	81% retention of the initial current	Repetitive CA tests over 30 days in 0.1 M NaOH containing 0.1 mM glucose	[47]
Activated carbon-supported Pt-Ni nanocomposite	89% retention of the initial current	Measuring changes in the anodic peak current in CV tests at +0.55 V in 0.1 M NaOH containing 0.3 mM glucose	[48]
PANI decorated with Nickel-Iron nanoparticles	90% retention of initial current	Repetitive CA tests over 30 days in 0.1 M NaOH and 1 mM glucose	[46]
PANI Nanofibers	Negligible changes in the redox current	Redox peaks from CV over 7 days in 0.1 M PB containing 2 mM glucose and 10 mM of ascorbic acid, uric acid, and lactate	[49]
Single-walled carbon nanotubes-porous silicon nanocomposites	95% retention of initial activity	CV responses recorded in PB over 25 days containing 2 mM glucose	[50]
Glassy carbon electrode modified with Ni_0.5_Cu_0.5_Co_2_O_4_ nanorods	No considerable changes	15 CV cycles in 1 mM glucose, 0.1 M NaOH	[51]
Graphene nanosheet modified with Nickel oxide	No considerable changes	Monitoring current over time for 10 days under a 100 Ω external load, in 0.1 M KOH containing 0.1 M glucose	[52]
Cobalt oxide hollow nanododecahedra	94.6% retention of the initial current	Repetitive CA tests 15 days in 0.1 M KOH with 0.5 mM glucose	[53]
Pt nanoparticles on graphene	>95% retention of initial current	CV tests for 20 days in 0.1 M PB (pH 7.4), 50 mM glucose	[54]
Polyaniline decorated with copper-nickel	89% retention of the initial current	Repetitive CA tests over 15 days in 1 mM glucose, 0.1 M NaOH	[55]
Pt_27_Pd_47_Cu_26_ alloy nanowires	No considerable changes	CA for 1 h in 0.1 M glucose, 0.5 M KOH	[56]
Polyimide/Au-polyaniline (PAN)/Pd	>98% retention of redox peaks	200 consecutive CV cycles in 10 µM glucose, 0.1 M NaOH	[57]

* unless specified, tests were performed at room temperature.

## Data Availability

Data are available upon request.

## Supplementary Materials

The following supporting information can be downloaded at: https://www.mdpi.com/article/10.3390/bios15090588/s1, Figure S1: Cyclic voltammogram of the hPG/Au electrode in 0.05 M H2SO4 solution at a scan rate of 50 mVs^−1^ for the calculation of the ESA; Figure S2: FESEM images of PANI/hPG/Au at increasing magnifications: ×1,000, ×10,000, and ×100,000, from right to left, respectively. All images were captured at an acceleration voltage of 5.0 kV. Figure S3: Chronoamperometric responses of (a) hPG/Au electrode at +0.24 V vs. Ag/AgCl (3 M KCl), (b) PANI/hPG/Au electrode at +0.29 V, and (c) PANI-Pt/hPG/Au electrode at +0.29 V generated to increasing concentrations of glucose in PB (0.1 M, pH 7.4). Figure S4. Assessment of stability under repetitive testing of current output generated by (a) hPG/Au electrode and (b) PANI/hPG/Au electrode through repetitive CA tests on the same device within the solution of 0.1 M PB containing 6 mM glucose. The repetitive tests were carried out using the same electrode, and each data point represents the mean of three replicates. Error bars show the SD from a minimum of three independent measurements (n ≥ 3) using different electrodes. Table S1: Analytical performance of the electrodes in higher concentration of glucose based on chronoamperometry.
