# Supplementary material for: Robust Pt/Au Composite Nanostructures for Abiotic Glucose Sensing
Source: Biosensors (Basel). 2025 Sep 8;15(9):588. doi: 10.3390/bios15090588 (PMC12467538; doi:10.3390/bios15090588)
Supplement: Supplementary file 1 [file biosensors-15-00588-s001.zip › biosensors-3829456-supplementary.pdf]

# Robust Pt/Au Composite Nanostructures for Abiotic Glucose Sensing

**Asghar Niyazi, Ashley Linden, and Mirella Di Lorenzo**

**SUPPLEMENTARY INFORMATION**

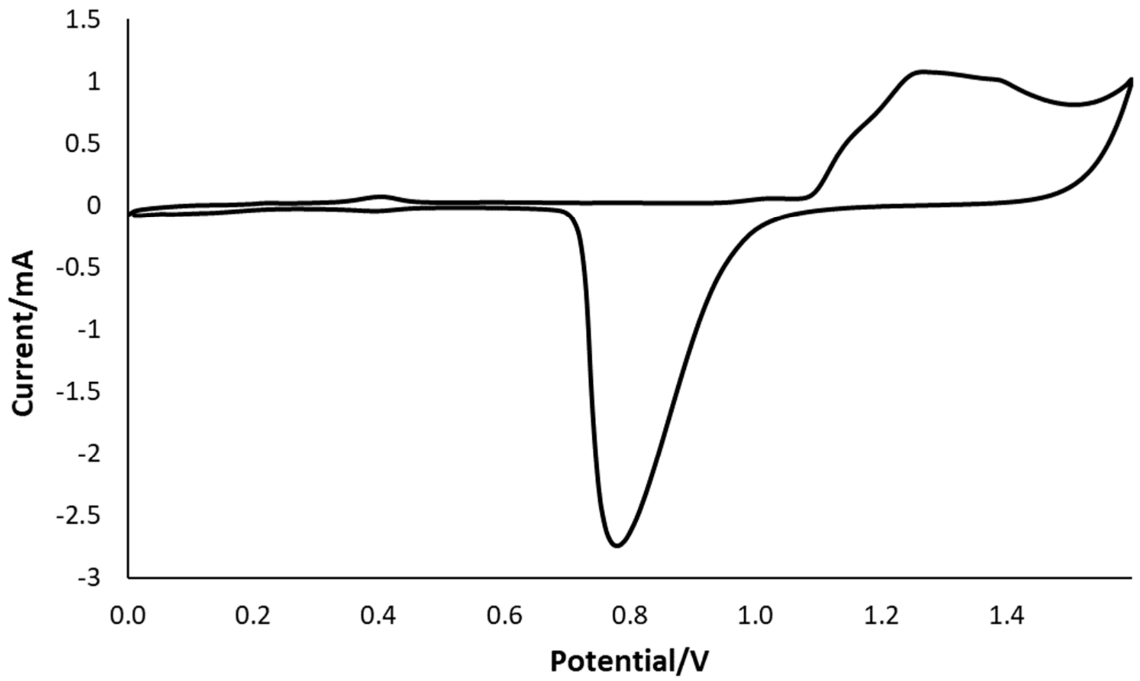


**Figure S1**. Cyclic voltammogram of the hPG/Au electrode in 0.05 M H2SO4 solution at a scan rate of 50 mVs-1 for the calculation of the ESA

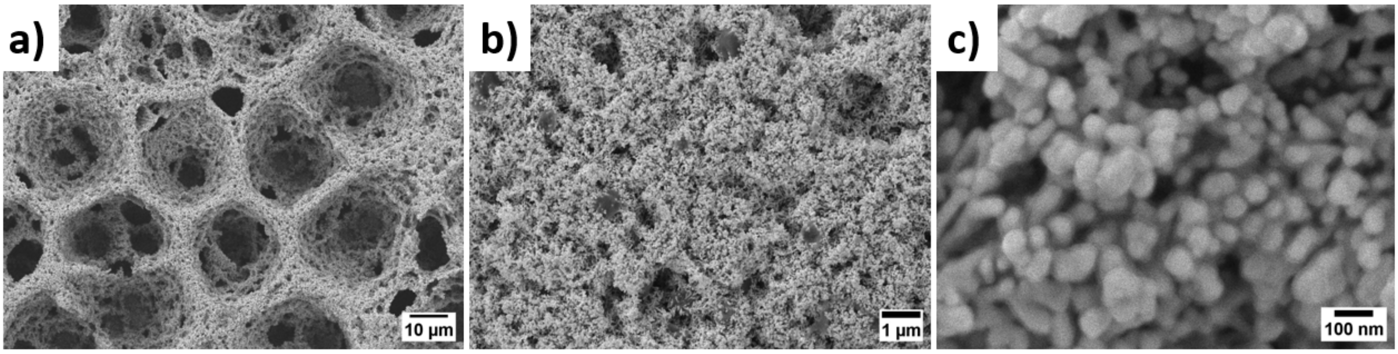


**Figure S2**. FESEM images of PANI/hPG/Au at increasing magnifications: ×1,000, ×10,000, and ×100,000, from right to left, respectively. All images were captured at an acceleration voltage of 5.0 kV.

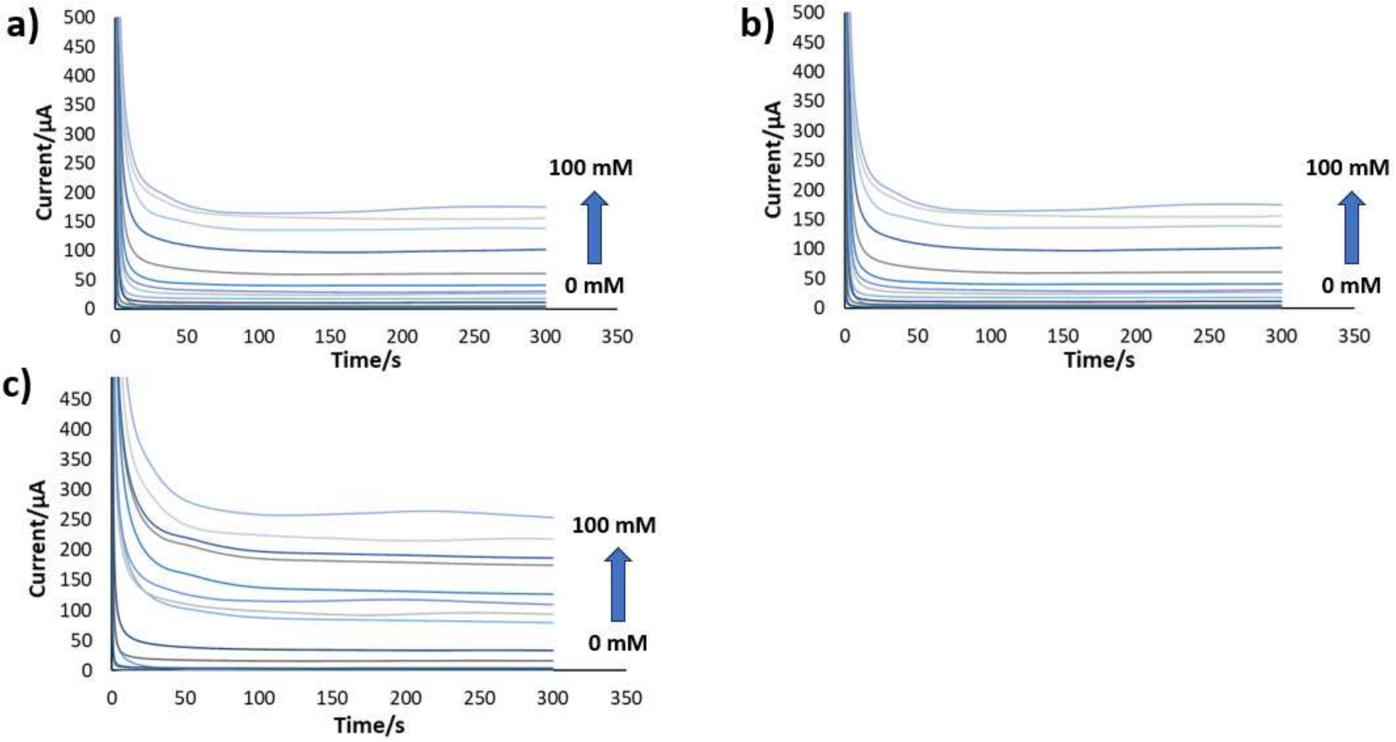


**Figure S3**. Chronoamperometric responses of a) hPG/Au electrode at +0.24 V vs Ag/AgCl (3 M KCl), b) PANI/hPG/Au electrode at +0.29 V, and c) PANI-Pt/hPG/Au electrode at +0.29 V generated to increasing concentrations of glucose in PB (0.1 M, pH 7.4).

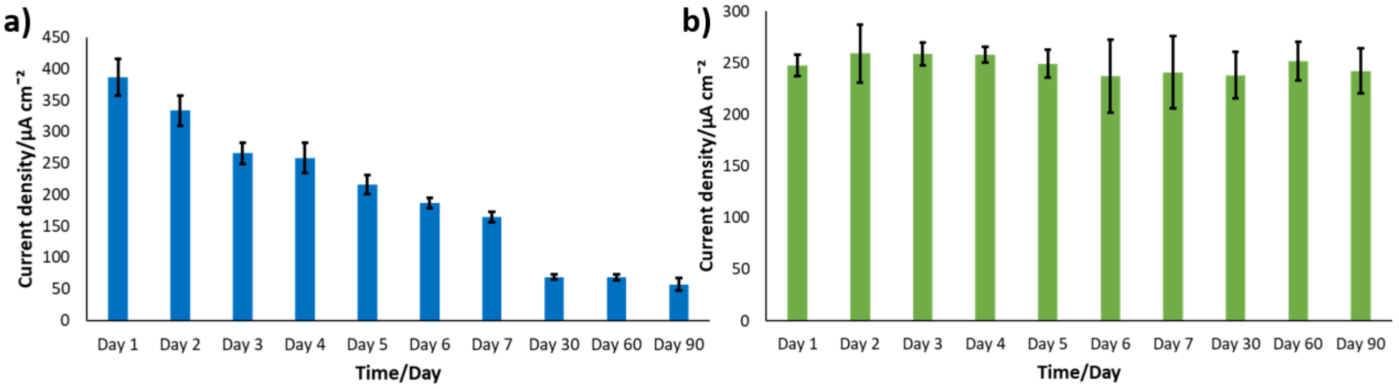


**Figure S4**. Assessment of stability under repetitive testing of current output generated by a) hPG/Au electrode and b) PANI/hPG/Au electrode through repetitive CA tests on the same device within the solution of 0.1 M PB containing 6 mM glucose. The repetitive tests were carried out using the same electrode, and each data point represents the mean of three replicates. Error bars show the SD from a minimum of three independent measurements (n ⩾ 3) using different electrodes.

**Table S1**. Analytical performance of the electrodes in higher concentration of glucose based on chronoamperometry.

| **Electrode** | **Linear Range 2 (mM)** | **Sensitivity 2 (μA $mM^{-1}$ $cm^{-2}$)** |
|---|---|---|
| PANI-Pt/hPG/Au | 30 – 100 | 12.87 ± 0.81 |
| PANI/hPG/Au | 60 – 100 | 12.35 ± 2.59 |
| hPG/Au | 50 – 100 | 21.03 ± 2.44 |
